# Vertical transmission and reproduction rate: modeling a common strategy for two related diseases

**DOI:** 10.1186/s40064-016-2096-6

**Published:** 2016-04-14

**Authors:** Abba Mahamane Oumarou, Yannick Tchaptchie Kouakep

**Affiliations:** Department of Mathematics, University Abdou Moumouni, P.O. Box 10 662, Niamey, Niger; University of Ngaoundere, P.O. Box 454, Ngaoundere, Cameroon; Department of Mathematics and Computer Science, AIMS - Cameroon, P.O. Box 608, Limbe, Cameroon

**Keywords:** Vaccination, Basic reproduction rate, Differential infectivity, 35Q92, 92D30, 92D25

## Abstract

Motivated by (Goyal and Murray in PLoS One 9(10):e110143, 2014) we consider a partially age-structured model simulating the dynamic of two infectious diseases vertically transmitted almost independently with horizontal coinfection and a common age-structured vaccination strategy. We study influence of parameters on existence and uniqueness of solutions and epidemiological equilibria. Impact of vertical transmission on basic reproduction rate is also presented.

## Introduction

This paper investigates influence of parameters on existence and uniqueness of solutions and equilibria in an age structured model. This model mimics the dynamic of two diseases vertically transmitted almost independently with horizontal coinfection and a common vaccination strategy. For example Goyal and Murray (2014) notes a decrease in Hepatitis B virus (HBv) prevalence as vaccination coverage increases and it is possible to eradicate both HBv and HDv (hepatitis D virus) using high vaccination coverage. Age structure *a* added to the continuous time *t* brings some improvements in the comprehension of the disease dynamics. For the case of HBv and HDv, age plays also a great role in the vaccination strategy (Goyal and Murray 2014). We follow methods of Djidjou et al. (2014), Yang et al. (2014), Brauer et al. (2013) or Inaba (1990) for quantitative (wellposedness with semigroup theory) studies. One can see also Castillo-Chavez and Feng (1998), Greenhalgh (2010) and references Hadeler and Muller (1996), Kouakep and Houpa (2014), Muller (1998, 2000), and Pasquini and Cvjetanović (1988) therein for a good review. Most of time in Africa (WHO 2014), vaccination campaigns concern more than one disease. This study starts with the case of two diseases and forthcoming works will deal with more than two diseases.

We study impact on basic reproduction rate (with a common vaccination strategy) of vertical transmission. Our goal is to bring our contribution with quantitative results concerning special cases in the context of non-linear dynamics of infectious diseases (in the context of the “Appendix 1”) and non local boundary conditions. We included vaccination ignored in Djidjou et al. (2014), vertical transmission neglected in Castillo-Chavez and Feng (1998) and show explicitly the basic reproduction rate theoretical shown with an equivalent number in term of asymptotic properties (threshold parameter) in Djidjou et al. (2014).

The paper is organised as follow: second, third and fourth sections are respectively devoted to problem formulations, primary material on the model, some asymptotic results with impacts on basic reproduction rate induced by vertical transmission. We conclude with a discussion and conclusions.

## Problem formulations

In this work we will consider the following model with vaccination for two diseases W and Y. (*s*, *v*, *I*, *E*, *R*) satisfies (see “Preliminary materials” section for Banach spaces used) the following system of equations:

1 $$\begin{aligned} \begin{aligned}&\left( \partial _t+\partial _a+\Psi (a)+\mu \right) s(t,a)=-\lambda (t)s,\;t,a>0,\\&\left( \partial _t+\partial _a+\mu \right) v(t,a)=\Psi (a)s(t,a)-\delta \lambda (t)v(t,a),\\&I'(t)=\lambda (t)\int _0^\infty p(a)\left( s(t,a)+\delta v(t,a)\right) da-\left( \nu _I+ \sigma -\Lambda _I\right) I(t),\\&E'(t)= \lambda (t)\int _0^\infty q(a)\left( s(t,a)+\delta v(t,a)\right) da-\left( \nu _E-\Lambda _E\right) E(t) + \sigma I(t),\\&R'(t)=-\mu R(t)+(\mu _I-\epsilon _1)I(t)+(\mu _E-\epsilon _2)E(t),\quad t>0, \end{aligned} \end{aligned}$$

posed for time $t>0$, (chronological) age $a>0$, $\mu _I,\mu _E,\mu ,\epsilon \ge 0$, $\mu _E\ge \mu _I$ with recovery rates $\mu _I-\epsilon _1\ge 0$ and $\mu _E-\epsilon _2\ge 0$. Here *s*(*t*, *a*) denotes the age-specific density of susceptibles to the two diseases, *E*(*t*) and *I*(*t*) denote respectively the age-specific densities of infected individuals by diseases Y and W (that can be symptomatic or asymptomatic) while *R*(*t*) denotes the recovered of both diseases and immunized individuals. *v*(*t*, *a*) is the density of vaccinated individuals for both diseases W and Y. We should note that disease W can make the infection by disease Y easier: that is why we see the possible transition $\sigma$ from compartment I to compartment E (see e.g. W $=$ hepatitis B and Y $=$ hepatitis D).

$\Lambda _I$ and $\Lambda _E$ traduce respectively the proportional influx of new infectives of W- and Y-types coming from vertical transmission (see “Appendix 1”). We will track their influences on basic reproduction rate.

To perform our analysis we shall assume that the contact between individuals is homogeneous so that $\beta _i(a,a')\equiv \beta _I>0{\text { and }}\beta _e(a,a')\equiv \beta _E\ge 0$ and vaccination strategies are time-independent $\Psi (t,a)\equiv \Psi (a) \ge 0$.

The term $\lambda (t)$ corresponds to the age-specific force of infection and follows the usual law of mass-action, that reads as $\lambda (t)=\beta _I I(t)+\beta _E E(t)$. Here $\beta _I$ and $\beta _E$ respectively denote the constant contact transmission rates between W-infected and Y-infected with all the susceptibles. $\Psi (t,a)$ is the proportion of susceptibles with age *a* vaccinated at time *t*. $0\le \delta \le 1$ is the reduction in risk due to prior exposure to vaccination (see Castillo-Chavez and Feng 1998). That means: $\delta =0$ corresponds to a perfect vaccine and $\delta =1$ corresponds to a totally imperfect vaccine. In addition $p\in L_+^\infty (0,\infty )$ is a given function such that $0\le p(a)\le 1$*a*.*e*. Function *q* (with $0\le q(a)\le 1$*a*.*e*.) represents the age-specific probability to become Y-infected when becoming infected by Y at age *a*. Function *p* denotes the probability to develop an W-infection when getting the infection W at age *a*. We refer for the exceptional case of hepatitis B to Edmunds et al. (1993) for more explanation on the age-dependence susceptibility to the infection and their estimations from data with maximum likelihood or least squares methods. This problem is supplemented together with the positive boundary conditions (choosing between $i=1$ and $i=2$):

2 $$\begin{aligned} \begin{aligned} s(t,0)&=f_i\left( \int _0^\omega \overline{\varepsilon }(a)\left[ s(t,a)+l_1 v(t,a)\right] da+l_2 R(t)+l_3 I(t)+l_4 E(t)\right) \\ s(0,a)&=s_0(a),\;v(t,0)=0,\;v(0,a)=v_0(a),\;I(0)= I_0,\; E(0)= E_0,\; R(0)= R_0\\ \end{aligned} \end{aligned}$$

where $\overline{\varepsilon }(a)=\left( 1-\varepsilon (a)\right)$ and $0\le \varepsilon (a) \le 1$, for a.e $a\ge 0$, $0\le \epsilon _1\le \mu _I$, $0\le \epsilon _2\le \mu _E$ and $l_1,l_2,l_3,l_4\in [0;1]$. We consider the fertility rate *f* as constant while defining the functions $f_1:x\in {\mathbb {R}}\mapsto f.x$ and $f_2:x\in {\mathbb {R}}\mapsto \Lambda \in [0;+\infty )$. Note that the *R* component of the system is decoupled from the other components and has therefore no impact upon the long time behaviour of the system. This model is derived from the age structured model presented in “Appendix 1”.

Like Republic of Niger’s or Cameroonian Governments (see Ministry 2014) we choose the situation with no newborn baby vaccination: $v(t,0)=0$. Technically in most part of this work, the maximum lifetime $\omega$ will be taken as $+\infty$ (through coefficients’ supports) for sake of simplicity.

## Preliminary materials

We point out that the case $i=2$ with $\sigma =0$ has been partially investigated in Kouakep and Houpa (2014) using integrated semigroup theory. We focus later on case $i=1$. In the sequel $\omega \in (0;+\infty ]$: it represents biologically the human maximum lifetime.

### Abstract formulation

Let *X* be the space defined as $X:=\left[ L^1\left( 0,\omega ;{\mathbb {R}}\right) \right] ^2\times {\mathbb {R}}\times {\mathbb {R}}\times {\mathbb {R}}$ endowed with the norm for $\varphi =\left( \varphi _1,\varphi _2,\varphi _3,\varphi _4,\varphi _5\right) ^T\in X$ with $\omega$ in $[0,+\infty ]$ and

3 $$\begin{aligned} \left\| \varphi \right\| _X:=\left\| \varphi _1\right\| _{L^1(0,\omega )}+\left\| \varphi _2\right\| _{L^1(0,\omega )}+\left| \varphi _3\right| +\left| \varphi _4\right| +\left| \varphi _5\right| \end{aligned}$$

Let $X^+$ be its positive cone (of *X*): $X^+:=\left[ L^1_+\left( 0,\omega ;{\mathbb {R}}\right) \right] ^2\times {\mathbb {R}}_+\times {\mathbb {R}}_+\times {\mathbb {R}}_+$ Let us note $X_+$ the positive cone of *X*. It is well known that $(X, \left\| .\right\| _X)$ is a Banach space (Djidjou et al. 2014). We define the linear operator $A:D(A)\subset X\rightarrow X$ defined by

4 $$\begin{aligned} \begin{aligned}&D(A)=\left\{ \varphi \in \left[ W^{1,1}(0,\infty )\right] ^2\times {\mathbb {R}}^3\right| \left. \begin{pmatrix} \varphi _1(0)\\ \varphi _2(0)\end{pmatrix} \equiv \begin{pmatrix} f^{**}(\varphi )\\ 0 \end{pmatrix}\right\} \end{aligned} \end{aligned}$$

together with $$f^{**}(\varphi ):=f_i\left( \int _0^\infty \overline{\varepsilon }(a)\left[ \varphi _1(a)+l_1 \varphi _2(a)\right] da+l_2 \varphi _5+l_3 \varphi _3+l_4 \varphi _4\right)$$ and

5 $$\begin{aligned} A\begin{pmatrix} \varphi _1\\ \varphi _2\\ \varphi _3\\ \varphi _4 \\ \varphi _5\end{pmatrix} \equiv \begin{pmatrix} -\varphi _1'(a)-\mu \varphi _1(a)\\ -\varphi _2'(a)-\mu \varphi _2(a)\\ -\left( \nu _I+\sigma -\Lambda _I\right) \varphi _3(a)\\ -\left( \nu _E-\Lambda _E\right) \varphi _4(a)\\ -\mu \varphi _5(a) \end{pmatrix}, \end{aligned}$$

as well as the nonlinear Frechet differentiable and locally Lipschitz map $F:\overline{D(A)}\rightarrow X$ defined by

6 $$\begin{aligned} \begin{aligned}&F\begin{pmatrix} \varphi _1\\ \varphi _2\\ \varphi _3\\ \varphi _4 \\ \varphi _5\end{pmatrix} = \begin{pmatrix} -\left( \beta _I \varphi _3+\beta _E \varphi _4+\Psi (a)\right) \varphi _1(a)\\ \Psi (a)\varphi _1(a)-\delta \left( \beta _I \varphi _3+\beta _E \varphi _4\right) \varphi _2\\ \left( \beta _I \varphi _3+\beta _E \varphi _4\right) \int _0^\infty p(a)\left( \varphi _1(a)+\delta \varphi _2(a)\right) da\\ \left( \beta _I \varphi _3+\beta _E \varphi _4\right) \int _0^\infty q(a)\left( \varphi _1(a)+\delta \varphi _2(a)\right) da+\sigma \varphi _3(a)\\ \left( \mu _I-\epsilon _1\right) \varphi _3(a)+\left( \mu _E-\epsilon _2\right) \varphi _4(a)\end{pmatrix}. \end{aligned} \end{aligned}$$

We set $X_0 := \overline{D(A)}$ and $X_{0+}$ the positive cone ($X_{0}\cap X^{+}$) of $X_{0}$. It is easy (see Djidjou et al. 2014) to see that if $\left( \nu _I+\sigma -\Lambda _I\right) \ge \mu$ and $\left( \nu _E-\Lambda _E\right) \ge \mu$, then:

1. the operator *A* satisfy Hille–Yosida property: $(-\mu ,\infty )\subset \rho (A)$ with
  7 $$\begin{aligned} \Vert (\lambda -A)^{-1}\Vert _{{\mathcal {L}}(X)}\le \frac{1}{\lambda +\mu },\;\;\forall \lambda >-\mu . \end{aligned}$$
  Moreover we have for each $\lambda >-\mu$: (1-) $(\lambda -A)^{-1}X^{+}\subset X_{0+}$;
2. *A* is generator of a $C_0$-semigroup of linear bounded operators;
3. the domain *D*(*A*) of operator *A* is dense in $X_0:=\overline{D(A)}$ and *A* is a closed operator.

Standard methodologies apply to provide (1) and (2) (see Pazy 1983) and (3) is due to the fact that the operator *A* is generator of a $C_0$-semigroup of linear bounded operators (see Corollary 2.5 in Pazy 1983). Therefore, one obtains that System (1)–(2) re-writes as the following densely defined Cauchy problem

8 $$\begin{aligned} \frac{d\phi (t)}{dt}=A\phi (t)+F(\phi (t)), \quad \phi (0)=\left( \varphi _1,\varphi _2,\varphi _3,\varphi _4,\varphi _5\right) ^T \end{aligned}$$

### Existence and uniqueness of solutions

For arbitrary $\phi _0\in X_{0+}$, we solve (8) as mild solution of the integrated equation (see Djidjou et al. 2014): $\phi (t)= \phi _0+A\int ^t_0 \phi (s)ds +\int ^t_0 F\left( \phi (s)\right) ds\;\;,\forall t\ge 0$. We obtain the following lemma.

#### **Lemma 1**

*Assume that*$\varepsilon \in L_{+}^{\infty }\left( 0,\omega ;{\mathbb {R}}\right)$, $\Lambda _I\le \epsilon _1$*and*$\Lambda _E\le \epsilon _2$, *then:*

*(a)*: *The operatorAis generator of a*$C_0$*-semigroup of linear bounded operators and the domainD*(*A*) *of operatorAis dense in*$X_0$*andAis a closed operator.*
*(b)*: *Moreover, the nonlinear operatorFfromXtoXis continuous and locally Lipschitz.*
*(c)*: (8) *generates a strongly continuous positive semiflow*$\{U(t)\}_{t\ge 0}$*on*$X_{0+}$. *This means that for each*$x=\phi (0)\in X_{0+}$, *the continuous map*$t\rightarrow U(t)x$*defined from*$[0,\infty )$*into*$X_{0+}$*is a weak solution of* (8), *that is (for the integrated problem)*
  $$\begin{aligned} \int _0^t \phi (s)ds\in D(A),\;\;\forall t\ge 0,\;\; \phi (t)=x+A\int _0^t \phi (s)ds+\int _0^t F\left( \phi (s)\right) ds,\;\;\forall t\ge 0. \end{aligned}$$
*(d)*: *It satisfies the following bounded-dissipative estimates for each*$x=\phi (0)\in X_{0+}$ (*with*$\omega =+\infty$*for sake of simplicity*) *and each*$t\ge 0$:
  9 $$\begin{aligned} \Vert x\Vert _X e^{-\overline{\Lambda } t}\le \Vert U(t)x\Vert _{X}\le \frac{\Vert x\Vert _X}{\underline{\Lambda }}\left( 1-e^{-\underline{\Lambda } t}\right) +\Vert x\Vert _X e^{-\underline{\Lambda } t}, \end{aligned}$$
  *or*
  10 $$\begin{aligned} \Vert x\Vert _X e^{-\overline{\Lambda } t}\le \Vert U(t)x\Vert _{X}\le \frac{\Vert x\Vert _X}{\mu }\left( 1-e^{-\mu t}\right) +\Vert x\Vert _X e^{-\mu t}, \end{aligned}$$
  *with*$$\underline{\Lambda }=\mu +min\left\{ \left( \epsilon _1-\Lambda _I\right) ,\left( \epsilon _2-\Lambda _E\right) \right\} ,$$*and*$$\overline{\Lambda }=\mu +max\left\{ \left( \epsilon _1-\Lambda _I\right) ,\left( \epsilon _2-\Lambda _E\right) \right\}$$.
*(e)*: *Theorem 1.4 in Pazy *(1983) *proves that for*$\phi _0\in X_{0+}=\left[ L^1_+\left( 0,\omega ;[0,+\infty )\right) \right] ^2\times [0,+\infty )^3$*there exists a unique bounded continuous solution*$\phi$*to the integrated problem defined on*$[0,+\infty )$*with values in*$X_{0+}$.

#### *Proof*

The proof of $[a-b-c-e]$ is rather standard. Indeed it is easy to check that operator *A* satisfies the Hille–Yosida property. Then standard methodologies apply to provide the existence and uniqueness of mild solution for System (1)–(2) (see for instance Djidjou et al. 2014, Pazy 1983 and the references therein).

We define the total population *P*(*t*) at time *t* by $P(t)=\int ^{+\infty }_0 \left( \phi _1(t,a)+\phi _2(t,a)\right) da+\phi _3(t)+\phi _4(t)+\phi _5(t)$ and use the fact that $P(0)=\Vert x\Vert _X$. The proof of [*d*] is immediate from the integration of the Eqs. (1)–(2) using formal differentiation of *P*(*t*) in respect to *t* and assumptions made: $\varepsilon \in L_{+}^{\infty }\left( 0,\omega ;{\mathbb {R}}\right)$, $\Lambda _I\le \epsilon _1$ and $\Lambda _E\le \epsilon _2$.$\square$

#### *Remark 1*

Under assumptions of Lemma 1, one could show that the semiflow $\{U(t)\}_{t\ge 0}$ is asymptotically smooth on *X* by using results derived by Sell and You (2002).

From Lemma 1 and above Remark 1, one deduces using the results of Hale (1989), Smith and Thieme (2011), and Magal and Zhao (2005) the following results:

#### **Lemma 2**

*Assume that*$\varepsilon \in L^{\infty }_+\left( 0,\omega ;{\mathbb {R}}\right)$, $\Lambda _I\le \epsilon _1$*and*$\Lambda _E\le \epsilon _2$. *The semiflow*$\{U(t)\}_{t\ge 0}$*provided by Lemma*1*has a non-empty compact global attractor*$\mathcal A\subset X_{0+}$. *It means that*$\mathcal A$*is compact, invariant and attracts all bounded set*$B\subset X_{0+}$, *such that for each*$B\subset X_{0+}$*bounded subset, one has*$d\left( U(t)B,\mathcal A\right) \rightarrow 0$*as*$t\rightarrow \infty$*whered*(*B*, *A*) *denotes the semi distance fromBtoAdefined by*$d(B,A)=\sup _{y\in B}\inf _{x\in A} \Vert y-x\Vert _X.$

## Asymptotic properties: impact on basic reproduction rate of vaccination efficiency and vertical transmission

In all this section we assume that $\varepsilon \in L^{\infty }\left( 0,\omega ;{\mathbb {R}}\right)$, $\Lambda _I<\epsilon _1$ and $\Lambda _E<\epsilon _2$. We will see that the basic reproduction rate $R_0$ is a decrease function of $\Psi$ as noticed by Goyal and Murray (2014).

We denote by $R^{\Psi }_0$ the basic reproduction rate with vertical transmission and vaccination for the new model (1)–(2). Then $R^{\Psi }_0:=R^{\Psi }_0(vert)$ has the value

11 $$\begin{aligned} \left[ \int _0^\infty \left( \frac{\beta _I p(a)}{a_1}+\frac{\beta _E q(a)}{a_2} + \frac{\beta _E \sigma p(a)}{a_3}\right) \times \left( s_F(a)+\delta v_F(a)\right) da\right] \end{aligned}$$

where $$a_1:=(\nu _I +\sigma -\Lambda _I)$$, $$a_2:=(\nu _E-\Lambda _E)$$ and $$a_3:=a_1 a_2$$.

### Steady states (DFE and EE)

Here we provide some information on steady states for (1)–(2).

#### **Lemma 3**

*Assume that*$\forall u\in \left\{ I,E \right\}$, $\Lambda _u < \nu _u$. *The following holds true:*

*(i)*: *If*$R^{\Psi }_0\le 1$, *then System* (1)–(2)-($i=1$) *has a unique stationary state*
  $$\begin{aligned} x_F=\left( s_F(a),v_F(a),0,0,0\right) ^T\in X_+ \end{aligned}$$
  *where*
  12 $$\begin{aligned} \begin{aligned} s_F(a)&=f_i\left( \int _0^\infty \left( 1-\varepsilon (u)\right) \left[ s_F(u)+l_1 v_F(u)\right] da\right) \\&\quad \times \,exp\left( -\mu a-\int ^{a}_0 \Psi (s)ds\right) ,\quad a\ge 0, \end{aligned} \end{aligned}$$
  *and*$$v_F(a)=\int _0^a \Psi (s)s_F(s)exp\left( -\mu \left( a-s\right) \right) ds$$.
*(ii)*: *If*$R^{\Psi }_0>1$, *then system* (1)–(2)-($i=1$) *has two stationary states: Disease free equilibrium (DFE)*$x_F\in X_+$*and Endemic Equilibrium (EE)*$x_E=\left( s_E(.), v_E(.),I_E, E_E, R_E\right) ^T$*with*
  13 $$\begin{aligned} \begin{aligned} s_E(a)&=f_i\left( \int _0^\infty \overline{\varepsilon }(s)\left[ s_E(s)+l_1 v_E(s)\right] ds\right. \\&\left. +\,l_2 R_E+l_3 I_E+l_4 E_E\right) \times exp\left( -\left( \lambda _E+\mu \right) a-\int ^{a}_0 \Psi (s)ds\right) ,\\ v_E(a)&=\int _0^a \Psi (s)s_E(s)exp\left( -\left( \delta \lambda _E+\mu \right) \left( a-s\right) \right) ds,\\ I_E&=\frac{\lambda _E}{\nu _I +\sigma -\Lambda _I}\int _0^\infty p(a)\left( s_E(a)+\delta v_E(a)\right) da,\\ E_E&=\frac{\lambda _E}{\nu _E-\Lambda _E}\int _0^\infty q(a)\left( s_E(a)+\delta v_E(a)\right) da +\frac{\sigma }{\nu _E -\Lambda _E}I_E\\ R_E&=\frac{(\mu _I-\epsilon _1)}{\mu }I_E+\frac{(\mu _E-\epsilon _2)}{\mu }E_E. \end{aligned} \end{aligned}$$
  *where*$\lambda _E>0$*is the unique solution of the equation*
  14 $$\begin{aligned} \begin{aligned} 1&=\int _0^\infty \left( \frac{\beta _I}{\nu _I+\sigma -\Lambda _I}p(a)+\frac{\beta _E}{\nu _E-\Lambda _E}q(a)\right. \\&\quad +\left. \frac{\beta _E\sigma p(a)}{(\nu _E-\Lambda _E)(\nu _I+\sigma -\Lambda _I)} \right) \left( s_E(a)+\delta v_E(a)\right) da \end{aligned} \end{aligned}$$

Rewriting Eq. (13) provides (see also Inaba 2001) a coupled integral equations system. The existence and uniqueness of continuous solutions $(s_E,v_E,I_E,E_E,R_E)$ for this type of Volterra like system is given by Gurtin and MacCamy (1974) (see a special case in “DFE special case (s*, v*, I* = 0, E* = 0, R* = 0): integral equation” and “EE special case (s*, v*, I* ≠ E* ≠ 0, R* ≠ 0): integral equation” sections in “Appendix 2”.

### Threshold number explained as basic reproduction rate

We recall $R^{\Psi }_0$ the basic reproduction rate with vertical transmission and vaccination for the model (1)–(2) (see “Appendix 1”). Clearly it is denoted $R^{\Psi }_0:=R^{\Psi }_0(vert)$ with the value

15 $$\begin{aligned} \left[ \int _0^\infty \left( \frac{\beta _I p(a)}{a_1}+\frac{\beta _E q(a)}{a_2} + \frac{\beta _E \sigma p(a)}{a_3}\right) \times \left( s_F(a)+\delta v_F(a)\right) da\right] \end{aligned}$$

where $a_1:=(\nu _I +\sigma -\Lambda _I),$ and $a_2:=(\nu _E-\Lambda _E)$ with $a_3:=a_1 a_2$. We recall the basic reproduction rate for our model with vertical transmission without vaccination

16 $$\begin{aligned} R^0_0(vert):=\left[ \int _0^\infty \left( \frac{\beta _I}{a_1}p(a)+\frac{\beta _E}{a_2}q(a) + \frac{\beta _E \sigma }{a_3}p(a)\right) s_F(a)da\right] \end{aligned}$$

and the basic reproduction rate for our model without vaccination and transition $\sigma=0$ nor vertical transmission

17 $$\begin{aligned} R^0_0(novert):=\left[ \int _0^\infty \left( \frac{\beta _I}{\nu _I}p(a)+\frac{\beta _E}{\nu _E}q(a)\right) s_F(a)da\right] \end{aligned}$$

or the basic reproduction rate $R^{\Psi }_0(novert)$ for our model with vaccination but vertical transmission and transition $\sigma=0$ excluded [see also Kouakep and Houpa (2014) with $\Lambda _E=0$, $\Lambda _I=0$ and $i=2$] is given by

18 $$\begin{aligned} \left[ \int _0^\infty \left( \frac{\beta _I}{\nu _I}p(a)+\frac{\beta _E}{\nu _E}q(a)\right) \left( s_F(a)+\delta v_F(a)\right) da\right] \end{aligned}$$

#### *Remark 2*

It is obvious that the vertical transmission increases the basic reproduction rate if $$\forall u\in \left\{ I,E\right\} ,\;\Lambda _u < \epsilon _{d(u)}\le \mu _u\le \nu _u$$ [more deaths than births, $$d(I)=1$$ and $$d(E)=2$$]. We focus on the case ($$i=1)$$.

#### **Theorem 1**

*Assume that* ( $\nu _E \le \Lambda _E$*or*$\nu _I+\sigma \le \Lambda _I$ ) *and*$h:=\mu +\nu _I+\nu _E-\left( \Lambda _I+\Lambda _E\right) > 0$. *Then System* (1)–(2)-$(i=1)$*has a unique stationary state (see integral equations in Gurtin and MacCamy *1974*; Krasnov et al.*^1977^*)*$x_F=\left( s_F(a),v_F(a),0,0,0\right) ^T\in X_+$*where*

19 $$\begin{aligned} \begin{aligned} s_F(a)&=f_i\left( \int _0^\infty \overline{\varepsilon }(u)\left[ s_F(u)+l_1 v_F(u)\right] du\right) \times e^{-\left( \mu a+\int ^{a}_0 \Psi (s)ds\right) } \end{aligned} \end{aligned}$$

$a\ge 0$*and*$v_F(a)=\int _0^a \Psi (s)s_F(s)exp\left( -\mu \left( a-s\right) \right) ds$. *Moreover, under its assumptions, results in Lemma*1*and Remark*1*on asymptotically smoothness of semiflow hold with this modification:*

20 $$\begin{aligned} \Vert x\Vert _X e^{-\overline{\Lambda } t}\le \Vert U(t)x\Vert _{X}\le \frac{\Vert x\Vert _X}{h}\left( 1-e^{-h t}\right) +\Vert x\Vert _X e^{-h t}, \end{aligned}$$

#### *Remark 3*

Authors like El-Doma (2006) choose to rewrite solution of the PDE (1)–(2) $(i=1)$ along characteristics and then construct a Lipschitz operator whose unique global in time fixed point will be the solution in Hadamard sense of the PDE.

## Discussion

The works of Castillo-Chavez and Feng (1998) and Djidjou et al. (2014) are more general by considering age-dependent death rates and birth fertility. But, our work connects these two important works in some of their complementary lacks and strength in order to study the impact on basic reproduction rate (with influence vertical transmission) of a common vaccination strategy inducing the stability of steady states of two related diseases. We saw that the basic reproduction rate $R_0$ is a decrease function of $\Psi$ confirming the decrease in Hepatitis B virus (HBv) prevalence as vaccination coverage increases (Goyal and Murray 2014): it is then possible to eradicate both HBv and HDv (hepatitis D virus) using high vaccination coverage. In further work we will include migrations in the infected individuals’ classes. One could biologically suspect the cases ($\nu _u \le \Lambda _u, \forall u\in \left\{ E,I\right\}$) in Lemma 1 to be critical since we would like to avoid blow-up of solutions in order to obtain global in time solutions. We said nothing in the cases: $\nu _E> \Lambda _E> \epsilon _2 {{{\text { or }}}} \nu _I+ \sigma> \Lambda _I > \epsilon _1$ What arises in Theorem 1 if $h=0$?

## Conclusions

The main objective of this work is study the impact of vertical transmission on basic reproduction rates in the case of coinfection like HBV(hepatitis B)/HDV(hepatitis D) co-infection. We found that vertical transmission increases the basic reproduction rate. Beside this, we studied the influence of the influx by migration on the wellposedness of the mathematical problem: there is a trade-off between entries balanced by mortalities and wellposedness for long term dynamic of our age-structured model. Some asymptotic relations between the mean of the fertility rate and other biological parameters are derived in endemic or free epidemic situations (“Appendices 1 and 2”). A perspective could be to introduce diffusion in our model and evaluate a minimal speed for travelling wave solutions.

## Appendix 1: Age structured model

Our model is derived (through $I(t):=\int _0^\infty i(t,a)da{\text {, }}E(t):=\int _0^\infty e(t,a)da$ and $R(t):=\int _0^\infty r(t,a)da )$ from:

21 $$\begin{aligned} \begin{aligned} \left( \partial _t+\partial _a+\Psi (t,a)+\mu \right) s(t,a)&=-\lambda _0(t,a)s(t,a),\\ \left( \partial _t+\partial _a+\mu \right) v(t,a)&=\Psi (t,a)s(t,a)-\delta \lambda _0(t,a)v(t,a),\\ \left( \partial _t+\partial _a +\left( \mu _I+\mu \right) \right) i(t,a)&=\lambda _0(t,a)p(a)\times \left( s(t,a)+\delta v(t,a)\right) - \sigma i(t,a),\\ \left( \partial _t+\partial _a+\mu _E+\mu \right) e(t,a)&=\lambda _0(t,a)q(a)\times \left( s(t,a)+\delta v(t,a)\right) + \sigma i(t,a),\\ \left( \partial _t+\partial _a +\mu \right) r(t,a)&=(\mu _I-\epsilon _1) i(t,a)+(\mu _E-\epsilon _2) e(t,a), \end{aligned} \end{aligned}$$

posed for time $t>0$, (chronological) age $a>0$, $\mu _I,\mu _E,\mu ,\epsilon \ge 0$, $\mu _E\ge \mu _I$ with recovery rates $\mu _I-\epsilon _1\ge 0$ and $\mu _E-\epsilon _2\ge 0$. Here *s*(*t*, *a*) denotes the age-specific density of susceptibles to the two diseases, *e*(*t*, *a*) and *i*(*t*, *a*) denote respectively the age-specific densities of infected individuals by diseases W and Y. The term $\lambda _0(t,a)$ corresponds to the age-specific force of infection reads as

$$\begin{aligned} \lambda _0(t,a)=\int _0^\infty \left[ \beta _i(a,a')i(t,a')+\beta _e(a,a')e(t,a')\right] da'. \end{aligned}$$

$\nu _I:=\mu _I+\mu$ and $\nu _E:=\mu _E+\mu$ and considering the fertility rate *f* as constant while defining the functions $f_1:x\in {\mathbb {R}}\mapsto f.x$ and $f_2:x\in {\mathbb {R}}\mapsto \Lambda \in [0;+\infty )$ with $w_{k,c}(t,a)=k.i(t,a)+c.e(t,a)$ and

22 $$\begin{aligned} \begin{aligned} s(t,0)&=\int _0^\infty f(a)b(a)\left( \overline{\varepsilon }(t,a)\left[ s(t,a)+l_1r(t,a)\right] +w_{1,1}(t,a)\right) da,\\ i(t,0)&=\Lambda _I \int _0^\infty i(t,a')da':\;\;w_{k,c}\equiv w_{1,0}\\ e(t,0)&=\Lambda _E \int _0^\infty e(t,a')da':\;\;w_{k,c}\equiv w_{0,1}\\ r(t,0)&=0,{\text { (no immunity at birth)}}, \end{aligned} \end{aligned}$$

The “sanitary” coefficient $\varepsilon (a) \in [0;1]$ describes the facility for an individual of age *a* to transmit vertically disease. If $\varepsilon =0$ the person can not transmit vertically the disease (he/she is “clean”). If $\varepsilon =1$ the person surely transmits vertically the disease (he/she is “totally infective”). $\overline{\varepsilon }(a)=\left( 1-\varepsilon (a)\right)$.

*f*(*a*) is the fertility rates while $b\in L_+^\infty (0,\infty )$ is a given function such that $0\le b(a)\le 1$, for a.e $a\ge 0$ and initial positive data $s(0,a)=s_0(a)$ and

23 $$\begin{aligned} \begin{aligned}&i(0,a)=i_0(a),\;\;e(0,a)=e_0(a),\;\; r(0,a)=r_0(a),\;\;v(0,a)=v_0(a). \end{aligned} \end{aligned}$$

*b*(*a*) is the specific probability to born susceptible.

## Appendix 2: Averaged fertility rate

### DFE special case $(s^*,v^*,I^*=0,E^*=0,R^*=0)$: integral equation

Straightforward computations lead for $a>0$ to:

24 $$\begin{aligned} \begin{aligned} s^*(a)&=f_i\left( \int _0^\infty \overline{\varepsilon }(u)\left[ s^*(u)+l_1 v^*(u)\right] du\right) exp\left( -\mu a-\int ^{a}_0 \Psi (s)ds\right) ,\\ s^*(0)&=f_i\left( \int _0^\infty \overline{\varepsilon }(u)\left[ s^*(u)+l_1 v^*(u)\right] du\right) ,\\ v^*(a)&=\int _0^a \Psi (s)s^*(s)exp\left( -\mu \left( a-s\right) \right) ds\\ v^*(0)&=0,\;\; E^*=I^*=R^*=0. \end{aligned} \end{aligned}$$

By setting $i=1$, $\varepsilon (u)=1-\alpha exp(-\gamma u),\forall u\ge 0$ and $l_1=1$ with $w^*:=s^*+v^*$, one obtains:

25 $$\begin{aligned} \begin{aligned} w^*(a)&=\int _0^\infty w^*(u) f(u)\left( 1-\varepsilon (u)\right) du \times exp\left( -\mu a\right) ,\;\;a>0,\\ w^*(0)&=s^*(0). \end{aligned} \end{aligned}$$

by setting a degenerated kernel (see Krasnov et al. 1977, p. 59–72) as

$$\begin{aligned} k(a,u)=\left( 1-\varepsilon (u)\right) exp\left( -\mu a\right) \end{aligned}$$

with some regularity on $\varepsilon$) or with $f(a)\equiv f\ge 0$

26 $$\begin{aligned} w^*(a)=f\int _0^\infty k(a,u) w^*(u) du,\;a>0,\quad w^*(0)=s^*(0). \end{aligned}$$

This is a second type homogeneous Fredholm equation. We have: $\int _0^\infty \int _0^\infty k(a,u)dadu$ finite. Krasnov et al. (1977) justify existence of solutions $w^*$ with associated characteristics value(s) *f*. By setting again (in the special case $f(u)\equiv f\ge 0$, $\varepsilon (u)=1-\alpha exp\left( -\gamma u\right) )\geq 0$, and $k(a,u)=\alpha exp\left( -\left( \gamma u+\mu a\right) \right)$ with $0\le \gamma,\alpha \le 1$ we get

27 $$\begin{aligned} \begin{aligned}&w^*(a)=f\int _0^\infty k(a,u)w^*(u)du,\;\;a>0,\\ \end{aligned} \end{aligned}$$

In fact, we could biologically and mathematically replace $\int _0^\infty \int _0^\infty$ by $\int _0^\omega \int _0^\omega$ with $\omega$ the maximal human admissible lifetime (e.g. $\omega =140\,{\mathrm{years}}$). Rewriting Eq. (24) along characteristics provides (see also Inaba 2001) a coupled integral equations system. The existence and uniqueness of continuous solutions $(s^*,v^*)$ for this type of Volterra like system is also given by Gurtin and MacCamy (1974).

Let consider the problem

28 $$\begin{aligned} \begin{aligned}&w^*(a)=f\int _0^\omega k(a,u)w^*(u)du,\;\;a>0 \end{aligned} \end{aligned}$$

where *f* is a characteristic value. We found $f=\left( \frac{\gamma +\mu }{\left( 1-e^{-(\gamma +\mu )\omega }\right) \alpha } \right)$ associated to the eigenfunction $m^*(a)=exp\left( -\mu a \right)$. If $\omega \rightarrow \infty$, then $f\rightarrow \left( \frac{\gamma +\mu }{\alpha }\right)$.

### EE special case $(s^*,v^*,I^*\ne 0,E^*\ne 0,R^*\ne 0)$: integral equation

Straightforward computations lead for $a>0$ to:

29 $$\begin{aligned} \begin{aligned} s^*(a)&=f_i\left( \int _0^\infty \overline{\varepsilon }(u)\left[ s^*(u)+l_1 v^*(u)\right] du\right) \times exp\left( -\mu a-\int ^{a}_0 \Psi (s)ds\right) ,\\ s^*(0)&=f_i\left( \int _0^\infty \overline{\varepsilon }(u)\left[ s^*(u)+l_1 v^*(u)\right] du\right) ,\\ v^*(a)&=\int _0^a \Psi (s)s^*(s)exp\left( -\mu \left( a-s\right) \right) ds\\ v^*(0)&=0,\quad E^*,I^*,R^*>0. \end{aligned} \end{aligned}$$

By setting $i=1$, $\delta \approx 1$, $\varepsilon (u)=1-\alpha exp(-\gamma u),\forall u\ge 0$ and $l_1=1$ with $w^*:=s^*+v^*$, one obtains:

30 $$\begin{aligned} \begin{aligned} w^*(a)&=\int _0^\infty w^*(u) f(u)\left( 1-\varepsilon (u)\right) du \times exp\left( -(\mu +\lambda _E) a\right) ,\quad a>0,\\ w^*(0)&=s^*(0). \end{aligned} \end{aligned}$$

by setting a degenerated kernel (see Krasnov et al. 1977, p. 59–72) as

$$\begin{aligned} k_1(a,u)=\left( 1-\varepsilon (u)\right) exp\left( -(\mu +\lambda _E) a\right) \end{aligned}$$

with some regularity on $\varepsilon$) or with $f(a)\equiv f\ge 0$

31 $$\begin{aligned} w^*(a)=f\int _0^\infty k_1(a,u) w^*(u) du,\quad a>0,\quad w^*(0)=s^*(0). \end{aligned}$$

Krasnov et al. (1977) justify existence of solutions $w^*$ with associated characteristic value(s) *f*.

By setting again (in the special case $f(u)\equiv f\ge 0$, $\varepsilon (u)=1-\alpha exp\left( -\gamma u\right) )$, and $k_1(a,u)=\alpha exp\left( -\left( \gamma u+(\mu +\lambda _E) a\right) \right)$ with $0\le \gamma \le 1$ we get

32 $$\begin{aligned} \begin{aligned}&w^*(a)=f\int _0^\infty k_1(a,u)w^*(u)du,\quad a>0,\\ \end{aligned} \end{aligned}$$

Let consider the problem

33 $$\begin{aligned} \begin{aligned}&w^*(a)=f\int _0^\omega k_1(a,u)w^*(u)du,\quad a>0 \end{aligned} \end{aligned}$$

where *f* is a characteristic value. We found $f=\left( \frac{\gamma +\mu +\lambda _E}{\left( 1-e^{-(\gamma +\mu +\lambda _E)\omega }\right) \alpha } \right)$ associated to the eigenfunction $m^*(a)=exp\left( -(\mu +\lambda _E)a \right)$. If $\omega \rightarrow \infty$, then $f\rightarrow \left( \frac{\gamma +\mu +\lambda _E}{\alpha }\right)$.
